# Evidence for Bulk Ripplocations in Layered Solids

**DOI:** 10.1038/srep33451

**Published:** 2016-09-19

**Authors:** Jacob Gruber, Andrew C. Lang, Justin Griggs, Mitra L. Taheri, Garritt J. Tucker, Michel W. Barsoum

**Affiliations:** 1 Department of Materials Science and Engineering, Drexel University, Philadelphia, PA 19104, USA.

## Abstract

Plastically anisotropic/layered solids are ubiquitous in nature and understanding how they deform is crucial in geology, nuclear engineering, microelectronics, among other fields. Recently, a new defect termed a ripplocation–best described as an atomic scale ripple–was proposed to explain deformation in two-dimensional solids. Herein, we leverage atomistic simulations of graphite to extend the ripplocation idea to bulk layered solids, and confirm that it is essentially a buckling phenomenon. In contrast to dislocations, bulk ripplocations have no Burgers vector and no polarity. In graphite, ripplocations are attracted to other ripplocations, both within the same, and on adjacent layers, the latter resulting in kink boundaries. Furthermore, we present transmission electron microscopy evidence consistent with the existence of bulk ripplocations in Ti_3_SiC_2_. Ripplocations are a topological imperative, as they allow atomic layers to glide relative to each other without breaking the in-plane bonds. A more complete understanding of their mechanics and behavior is critically important, and could profoundly influence our current understanding of how graphite, layered silicates, the MAX phases, and many other plastically anisotropic/layered solids, deform and accommodate strain.

Layered materials are ubiquitous in nature; they are present in geological formations, nuclear reactors, microcircuits, sensors, biomaterials and polymers-both natural and synthetic-and are important players in the nanomaterials revolution. Herein, layered solids are defined as materials that exhibit strong plastic anisotropy, more specifically where deformation is confined to two dimensions. In hexagonal solids with high c/a ratios, such as the MAX phases and many ionic ceramics, the energy of formation of non-basal dislocations is prohibitive, confining deformation to the basal planes. Another important class of layered solids are those with low c_44_ values as a result of weak-typically van der Waals (vdW)-interlayer bonding. These include graphite, ice, clays, transition metal dichalcogenides, double metal hydroxides, and MXenes among others.

The deformation of layered materials has been studied extensively for decades, especially layered silicates in geology. Deformation through kink bands (KBs) is commonly observed in layered solids, such as mica^1^, graphite^2^, ice^3^, and the MAX phases^4^. KBs have been observed in micas/biotites, at many scales^5^,^6^,^7^,^8^. It has also long been assumed that layered silicates, ice, the MAX phases, and graphite, etc. deform predominantly by the nucleation and slip of basal dislocations (BDs)^1^,^2^,^3^,^7^,^8^,^9^. However, many aspects of the deformation of layered materials remain poorly understood. Specifically, many layered solids have been classified as kinking nonlinear elastic (KNE), including the MAX phases^4^, mica^10^, graphite^11^ and many ionic ceramics^12^,^13^. KNE solids are characterized by fully and spontaneously reversible stress-strain loops when polycrystalline samples are compressed^14^,^15^, or single crystals are nanoindented (Supplementary Information Fig. 1a)^16^,^17^. Deformation in these materials also frequently produces local delaminations, though the presumed deformation mechanisms fail to account for c-axis strain (Supplementary Information Fig. 1b).

Based on early work by Frank and Stroh^18^, Barsoum *et al*. proposed that incipient kink bands (IKBs), opposite signed dislocation walls, can explain KNE behavior^19^. More recently the bowing of dislocations in kink boundaries (KBs) has been suggested as a possible mechanism for KNE behavior in hexagonal metals^20^. However, while the latter probably explains energy dissipation in hexagonal metals^16^, it falls short in explaining the behavior exhibited by the MAX phases–and by extension other KNE solids–in response to compressive stresses. The most glaring is the inability of BDs to account for clear evidence of c-axis strain^17^.

Recently, the possibility of another micromechanism was suggested by Kushima *et al*.^21^, who coined the term *ripplocation* to describe the buckling of surface layers in MoS_2_-a vdW layered solid-in response to mechanical loading. This defect is related, but distinct, from intrinsic rippling of 2D materials^22^ or that induced by defects^23^,^24^. A computational treatment of ripplocations^21^ showed that their energy scales sub-linearly with size and that-in sharp contradistinction to dislocations-identical surface ripplocations attract and combine^21^. In this work we extend the idea of ripplocations to two KNE solids: Ti_3_SiC_2_ and graphite.

The MAX phases, our experimental material for this work, are layered, ternary, hexagonal, early transition metal carbides and nitrides with the chemical formula, M_n+1_AX_n_, where M is an early transition metal, A in an A-group element (mostly groups 13 and 14) and X is carbon and/or nitrogen and n ranges from 1 to 3^4^. These phases are well-suited to study ripplocations because the mostly metallic character of their bonding keeps the atomic layers from fragmenting or fracturing when compressed^4^.

Graphite, another KNE solid^11^,^25^, is a component of current and future nuclear reactors^26^,^27^. Despite extensive investigation, its response to displacive irradiation remains an area of active study^28^,^29^,^30^. As detailed by Heggie *et al*.^31^, existing models of graphite do not explain why or how, upon irradiation, pyrolytic graphite: (i) shrinks parallel to, and expands normal to, the basal planes and, (ii) spontaneously forms KBs^32^. The same group suggested that c-axis expansion may arise from a defect described as a ‘ruck and tuck’ that originates from buckling of individual layers^31^.

The purpose of this paper is threefold. The first is to make the case that *bulk* ripplocations (BRs) exist in layered solids, that is, they are neither confined to vdW solids or near surfaces. The second is that they are a fundamentally different from BDs and thus constitute a new micromechanism in the deformation of solids. The last is to propose a framework in which ripplocation-mediated deformation may produce kinking non-linear elastic behavior. To make our case, we performed atomistic simulations on graphite and show direct transmission electron microscope, TEM, evidence for BRs in Ti_3_SiC_2_ grains that were deformed under a spherical nanoindenter. Ideally, it would have been best to computationally model and show TEM results on the same material. This was not possible here because the appropriate interatomic potentials for the MAX phases have, to date, not been developed precluding atomistic calculations. In addition, the difficulty in preparing graphite samples for TEM observation is well known^33^. However, since both graphite and Ti_3_SiC_2_ are KNE solids, we believe our approach is valid.

To study the behavior of individual ripplocations in our computational framework, single graphite atomic layers were axially compressed, at 10 K, by displacing their ends inwards, along the x-axis in Fig. 1. Following the methodology of Kushima *et al*.^21^, ripplocation size is characterized by *n*, the excess material within a layer, defined as the x-axis unit translation vector equal to the *a*-lattice parameter of graphite. Four different configurations were examined with atomistic simulations. Surface ripplocations (SRs) were nucleated by: (i) straining the top layer in a bilayer (not shown) or, (ii) by straining the top layer in a 16-layer system (Fig. 1a–d). In both cases, the bottommost layer was held immobile. BRs were nucleated by, (iii) straining an arbitrary layer within a 60 layer periodic system in the z-axis (Fig. 1e–h) or, (iv) by straining the middle layer in a tri-layer configuration, where the two outer layers are not allowed to move (Fig. 1i–k). These four configurations will henceforth be referred to as: (i) constrained SR, (ii) unconstrained SR, (iii) unconstrained BR, and, (iv) constrained BR, respectively.

In all configurations, the strained layer deformed, initially by elastic in-plane strain (along x in Fig. 1), up to a critical strain, ε_cr_, beyond which it spontaneously buckled to form ripplocations (Supplementary Information Movie 1). In all cases, buckling is associated with an energy reduction (Supplementary Information Movie 1e). Discrete localized ripplocations nucleated in both surface configurations (Fig. 1a–d and Supplementary Information Movie 1a,b) and the unconstrained BR (Fig. 1e–h and Supplementary Information Movie 1d). In the constrained BR case, diffuse buckles-spanning the simulation cell width-were produced (Fig. 1i–l and Supplementary Information Movie 1c). From these results, it is clear excess material, *n*, needed to nucleate the SRs, is the lowest, followed by that of the unconstrained BRs; the constrained BRs are the most difficult to nucleate. Note that the ripplocation nucleation barrier must fall between the two extremes modeled here: an unconstrained free surface (Fig. 1a) and a totally constrained system (Fig. 1i).

With increasing strain, SRs and unconstrained BRs continually grow to form more complicated folded structures (Fig. 1d,h). The folded BR shown in Fig. 1h is identical to the ruck and tuck defect proposed by Heggie *et al*.^31^. The SRs (Fig. 1a–d) closely match the one obtained in *ab initio* calculations in MoS_2_^21^, strongly suggesting the two defects are manifestations of the same mechanism. Importantly, the nucleation of ripplocations, not just in *ab initio* calculations, but also in our simpler, atomistic framework, indicates that nucleation does not require fine resolution of complex electronic interactions, but is primarily a buckling phenomenon. These results agree with Liu *et al*.^34^ who recently concluded that kinks they observed in graphite were due to buckling, and with Klaver *et al*.^35^ who used atomistic simulations to explain why when graphene layers-deposited on Cu at high temperatures-were cooled, single surface wrinkles, that were quite mobile, formed as a result of buckling.

Beyond ε_cr_, the energy of the discrete ripplocations per unit length, E_r_, no longer depends on strain, a function of total system size, but only on *n* (Fig. 2a). Differences in E_r_ between the unconstrained and constrained SRs are small. Beyond ε_cr_, fits (shown in Fig. 2a by dashed red lines) show E_r_ ∝ n^α^, with α = 0.44 and 0.48 for the unconstrained and constrained SRs configurations, respectively, closely matching the 0.4 value reported for MoS_2_^21^.

In bulk, the unconstrained (Fig. 1e–h) and constrained (Fig. 1i–l) configurations differ radically. In the latter, delocalized ripples–spanning the cell’s width-form as the immobile layers prevent the formation of large, single buckles (Supplementary Information Movie 1c). Consequently, after the onset of buckling, E_r_ scales super-linearly with *n* (Fig. 2a) but is still lower than the in-plane elastic response. Conversely, E_r_ for unconstrained BRs (solid blue diamonds in Fig. 2a) is sub-linear and does not conform to the aforementioned scaling law, probably because of the transition to more complex folded structures (Fig. 1h). This transition occurs with less excess material compared to SRs (n = 3 vs. n = 4), likely because the transition also reduces the long-range strain field in the surrounding layers, resulting in significantly higher E_r_ values for BRs than SRs.

The stark contrast between constrained and unconstrained BRs implies that the ability of the surrounding layers to deform out of plane is critical. Kushima *et al*. showed that it is favorable for SRs to agglomerate into larger buckles^21^, and while this is also true in the bulk, the tendency is modulated by the energy penalty required to strain the surrounding lattice. Consequently, a competition between large discrete buckles (Fig. 1a–d), with diffuse elastic fields (Fig. 1j–l) and deformable surrounding layers, is always present (Fig. 1e–h). Graphite falls in the latter category; with increasing *n*, the size of the BRs increases, but their number remains constant (Fig. 1e–h).

To begin quantifying any differences between ripplocations and dislocations, a comparison of their Burgers vectors is performed. The Burgers vector, ***b***_i_, can be directly computed using the integral


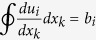


where ***u*** is the displacement vector and ***x*** is the position vector^36^. For circuits drawn far from the dislocation core, the value of this integral converges to the dislocation’s Burgers vector **b**_i_. In sharp contrast, Fig. 2b demonstrates that as circuit size increases-beyond a circuit radius of ≈ 40 Å-**b**_i_ for a BR in graphite goes to zero.

The absence of a Burgers vector has several implications. Defects with non-zero Burgers vectors have an intrinsic sign that informs defect-defect interactions. For instance, same-signed dislocations repel, while oppositely signed dislocations attract. Ripplocations, lacking a Burgers vector, have no intrinsic sign. This suggests that the interaction of one ripplocation with another will be invariant with regard to reflections of individual ripplocations. In Movie 2a, the BRs are identical; in Movie 2b the second ripplocation has been reflected over the x-axis. In both situations, however, the BRs attract. The lack a Burgers vector also suggests a better description for BR behavior can be found not in individual dislocations, but in dislocation complexes with a total Burgers vector of 0, such as small interstitial loops. Both of these defects represent an excess of material compared to a perfect crystal lattice. Interestingly, the sub-linear energy scaling of unconstrained BRs with *n* is reminiscent of that of interstitial loop behavior in metals^37^,^38^. However, a direct comparison of the energetics of interstitial loops and BRs in graphite (Supplementary Information Fig. 2) shows that, at small *n*, the latter are energetically favored, since they lack the high energy cost associated with dangling C-C bonds. For example, our atomistic calculations show that the energy, ∆E, of an interstitial loop in graphite drops mildly from 16.5 to 16 eV as *n* increases from 1 to 4. In the same interval, ∆E of a BR increases from 1.5 to ≈4 eV. At very large *n*, as the dangling bond energy is amortized over an increasingly large number of atoms, it is likely that interstitial loops are energetically favored and large ripplocations may collapse into interstitial loops. Note that since both interstitial loops and BRs accommodate extra material, their long-range behavior may be quite similar. The core structures of these defects, however, differ substantially. It is possible that dislocation complexes with a total Burgers vector of 0 may explain some ripplocation behavior, but it is unlikely that they can predict certain ripplocation properties, notably the spontaneous reversibility on the removal of load (see below).

To obtain direct TEM evidence for the existence of BRs we performed spherical NI experiments on the MAX phase, Ti_3_SiC_2_. Select (0001) and (10
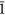
0) planes in a coarse-grained Ti_3_SiC_2_ polycrystalline sample were nanoindented into the same location 50 times. Bright- and dark-field TEM images of a Ti_3_SiC_2_ region far from the indented regions showed few defects (Supplementary Information Fig. 4a,b). In contrast, Fig. 3a shows a typical area beneath the indenter–loaded normal to the basal planes-where multiple line of dark contrast run parallel to the basal planes. Similar features were obtained when the basal planes were loaded edge-on (Supplementary Information Fig. 4c). These dark areas show mottled contrast-reminiscent of mottled contrast type 1 described by Noe and Veblen^39^,-in weak-beam dark field images, (Supplementary Information Fig. 4d) and were therefore chosen for high-resolution TEM (HRTEM) analysis. When observing these areas at higher magnification, (Fig. 3b) an area of dark phase contrast where the mottled contrast existed in weak-beam images is clearly visible. This dark phase contrast is due to the local lattice distortion along the basal planes. The inverse fast Fourier transform (IFFT) of the (0001) basal planes (Fig. 3c) highlights these distortions that appear as undulations. Employing geometric phase (GPA) analysis^40^, strain (Fig. 3d) and rotation maps (Fig. 3e) of the local lattice were created and confirmed the absence of dislocations in this area. This is clearly shown in the c-axis strain map, Fig. 3d, which shows a region of c-axis expansion surrounded by areas of compression. Non-basal dislocations have never been observed in the MAX phases and therefore, these lattice distortions cannot result from non-basal dislocations. It is worth noting that the GPA analysis was performed using *g* = (0001) and (11
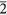
0).

The basal plane rotation map, Fig. 3e, obtained from GPA of the (0001) reflection shows an approximate 1–1.5° rotation of the basal plane within the dark regions, revealing strong evidence for basal plane rippling within this region. Leveraging atomic strain metrics^41^,^42^,^43^, Fig. 3f presents the c-axis strain field associated with an unconstrained BR with n = 3 in graphite. Similar to the strain field obtained from analysis of the HRTEM images (Fig. 3d), alternating regions of compressive and tensile strain are observed along the basal planes, with a central region of large compressive strain. Furthermore, the micro-rotation field surrounding this BR (Fig. 3g) is strikingly similar to those shown in Fig. 3e; opposite lobes of equal and opposite rotations at right angles to each other. The presence of only two lobes suggests that the experimental defect structure is not exactly identical to the defect produced in simulation, but likely shares structural components. This is not too surprising given the diversity of BR structures produced in the simulations, especially at higher n values. The numerical values of the strain field cannot be compared directly since the experimental and computational studies used different materials and reference states, but the two fields are qualitatively similar, suggesting that BRs provide an explanation for this unknown, but ubiquitous defect. It is crucial to appreciate that no g.b condition was found under which defects, such as the one shown in Fig. 3b, were rendered invisible in the TEM. This is further strong evidence that what we see is not a basal dislocation.

A key feature of BRs is that they tend to agglomerate, not only when present on the same layer, but also when present on adjacent layers (Supplementary Information Movie 2c,d). By combining in this manner, BRs provide a potential pathway for axial compressive strains to produce KBs. In order to visualize how and under what conditions BRs would nucleate we carried out a large-scale atomistic simulation of edge-on indentation of graphite with a cylindrical indenter (Supplementary Information Movie 3). Here, the application of load induces the spontaneous formation of oppositely oriented KBs along the basal plane direction, comprised of BRs (Fig. 4a). Upon the removal of the load, the system returns to its initial pristine state; the process is both dissipative and fully reversible. Based on the nucleation and evolution of BRs in this simulation, it is conceivable that at higher strains with a spherical nanoindenter, permanent KBs that could lead directly to the delamination-normal to the c-axis-observed in Fig. 4b might nucleate. Note that the atomistic simulation shown here was not performed with conditions that would allow delamination.

Solids that exhibit KNE behavior can easily be identified by the type of damage produced after nanoindentation. When KNE solids are loaded edge-on, after the load is removed–and despite the dissipation of significant amounts of energy–there is almost no trace of an indentation mark^17^. At larger loads, indentation marks are characterized by delamination cracks when loaded edge-on^17^, and by large craters and pile-up features when loaded normal to the planes (Supplementary Information Fig. 3a,b). In contrast, when other KNE solids, such as ZnO^44^ are loaded edge-on or Mg single crystals, are indented normal to the basal planes^16^, classic, smooth indentation marks (Supplementary Information Fig. 3c,d) are observed. This is likely due to nucleation of partially mobile <c + a> dislocations that, in turn, allow plastic strain normal to the basal planes. Interestingly, if twins nucleate, the propensity for delaminations and pileups is greatly suppressed^11^,^45^. It follows that spherical nanoindentations provide a convenient method for identifying materials in which ripplocations may be active: if indentation marks exhibit delamination cracks and large pile-up features, ripplocations may be active.

Based on the foregoing arguments, BRs can potentially explain a number of observations that to date have not been understood. Specifically, BRs offer a mechanism for the formation of KBs and the dissipation of significant amounts of energy per cycle both when loaded edge-on (B loops in Supplementary Information Fig. 1a) or normal to (A loops in Supplementary Information Fig. 1a) the basal planes. It is important to note that when indented normal to the basal planes, the nucleation of BRs requires the breaking of in-plane bonds. That such rupturing occurs is clearly seen in indentation marks in graphite and Ti_3_SiC_2_ that show large pileup features near the indentation edges (Supplementary Information Fig. 3a,b). Strikingly, in the case when the basal planes were loaded edge-on, after the indenter is removed–and despite the dissipation of significant amounts of energy during each cycle (Supplementary Information Fig. 1a)–there is almost no trace of an indentation mark^17^. It is not unreasonable at this stage to assume that what occurs in this case, is fundamentally similar to what our simulation suggests (see Supplementary Information Movie 3).

In conclusion, atomistic simulations confirmed that the behavior of discrete ripplocations is not dependent on the total system size (i.e., strain), but only on the amount of additional material added per plane. They also revealed that, unlike SRs, the behavior of BRs is strongly dependent on the ability of surrounding layers to deform; pliable layers give rise to large, single ripplocations that eventually transition to folded structures, while stiff layers create diffuse, non-localized buckles. When individual BRs form they provide a lower energy configuration for accommodating extra material as compared to in-plane strain even in the most constrained case. The Burgers vector of an isolated ripplocation is calculated to be 0, suggesting that ripplocations have no polarity consistent with the fact that BRs attract in graphite regardless of their polarity (see Supplementary Information Movie 2a,b). Lastly, it is crucial to point out that ripplocations are a topological imperative, as they allow atomic layers to glide relative to each other without breaking the in-plane bonds.

BRs are observed in HRTEM of mechanically deformed Ti_3_SiC_2_ crystals. The strain of individual atomic layers in graphite performed by atomistic simulations also produces localized buckling producing strains and rotations qualitatively similar to those observed in the TEM. This strongly suggests that the defects observed in Ti_3_SiC_2_ and BRs produced in our atomistic modeling are created by the same mechanism. Ti_3_SiC_2_ and graphite are both layered, KNE solids so their fundamental deformation mechanisms should be the same.

Simulation of the nanoindentation of graphite revealed that plane buckling is a key mechanism that not only accommodates c-axis strain but also produces fully reversible stress-strain hysteresis loops. Quantifying this response is a direction of future work. Future work should also investigate the interactions between BRs and point defects and whether or not there is an influence on their mobility. Work on other layered solids with different interlayer potentials should also be simulated. As noted above, to perform this work on the MAX phases require the development of accurate interatomic potentials. Another important question not investigated by this work is the behavior at the ends of finite length ripplocations or their junctions/nodes, which may exhibit complicated reconstructions that can substantially change their energetics.

## Methods

### Atomistic Simulation

Atomistic simulations were performed using the LAMMPS molecular dynamics, MD, package^46^ using the AIREBO inter-atomic potential^47^ for carbon and hydrogen, which includes both covalent and Van der Waals bonding between C atoms and is therefore well suited to modeling of graphite^48^. A Lennard-Jones cutoff scale factor of 4, equivalent to a cutoff radius of 13.6 Å was employed.

Compressive strain was generated in individual atomic layers by displacing one of the ends inwards. A group of four graphite unit cells at either end was fixed rigid, and one of the ends was displaced inwards by 2% of the x-axis translation vector every step, followed by conjugate gradient minimization. This process was performed until the total displacement was equal to 4 times the translation vector.

**Figure 1:**
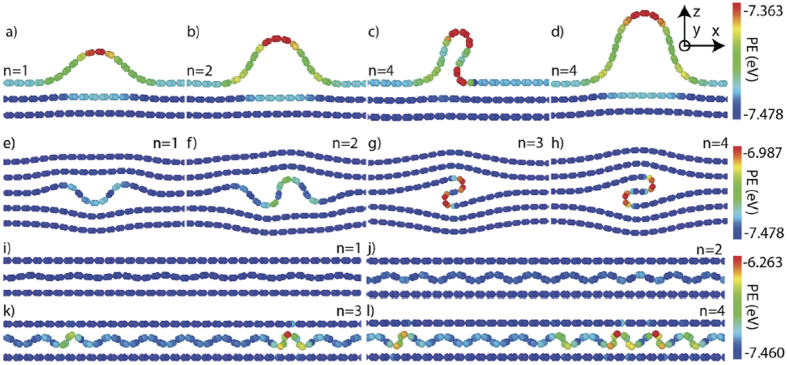
Core structures of ripplocations as a function of n: (**a**–**d**) unconstrained surface showing two forms of n = 4 structure; (**e**–**h**) bulk unconstrained and, (**i**–**l**) bulk constrained. Atoms are colored according to their potential energy defined from the interatomic potential whose scale is shown on the right.

To study the behavior of individual ripplocations, a single atomic layer was axially compressed in four different configurations. The four configurations were: (i) Surface ripplocations (SRs) nucleated by straining the top layer in a bilayer (not shown), (ii) SRs, nucleated by straining the top layer in a 16-layer system (Fig. 1a–d). In this figure, only the topmost 3 layers are shown. In both cases, the bottom layer was held immobile. (iii) Bulk ripplocations (BRs) nucleated by straining an arbitrary layer within a system of 60 layers periodic in the z-axis (Fig. 1e–h). In this figure only 5 layers of the 60 are shown, and, (iv) BRs nucleated by straining the middle layer in a tri-layer configuration (Fig. 1i–k). In this configuration, the two outer layers are fixed and rigid.

Unless otherwise specified, all configurations were periodic in the y-axis only. All configurations spanned 20 unit cells in the x-axis and 2 unit cells in the periodic y-axis. At displacements corresponding to integer multiples of the unit cell width, i.e. *na*, systems were trimmed and made periodic in the x-axis and underwent repeated cycles of conjugate gradient minimization and 1 ps of low-temperature (10 K) molecular dynamics simulation in order to obtain the true ground state.

The interaction of BRs was examined by replicating these minimized configurations, performing affine transformations and combining cells, taking care not to disrupt the arrangement of C atoms in the layers. Strain and rotation maps were produced using atomic scale kinematic metrics^41^. Results were visualized using OVITO^49^.

The simulated nanoindentation was performed using a cylindrical 20 nm radius indenter moving sinusoidally with a period of 500 ps and amplitude of 20 nm. Periodic boundary conditions were enforced along the y and z-axes. A thin region opposite the indenter, on the x-axis, was held fixed.

The Burgers vectors were estimated by calculating the displacement of each atom from a planar-layered reference configuration. A continuum displacement field was interpolated from the atomic displacements, allowing the construction of arbitrary Burgers circuits over which the Landau-Lifshitz integral was computed.

### Sample Synthesis and Preparation

A highly oriented coarse-grained (CG) Ti_3_SiC_2_ bulk sample was used in this study. The sample was synthesized by mixing Ti (99.9% metal basis, Alfa Aesar), Si and C (Alfa Aesar) elemental powders in stoichiometric ratios and hot pressing at 1450 °C, under a stress of 25 MPa for 4h. To coarsen the grains, the sample was annealed at 1550 °C for 72 h in Ar resulting in average grain sizes 200–500 μm. The sample was mostly pure, with only trace amounts of SiC present. The sample was mounted onto an Al mount, ground using 600–1200 SiC grit paper and polished down using a water-based suspension. Finally, the sample was vibropolished to 0.05 μm diameter sized particles using an alumina suspension. A scanning electron microscope (SEM) equipped with electron backscatter diffraction (EBSD) was used to generate an OIM map of the resultant microstructure, indicating a grain size on the order of 200–500 μm. A grain having a (0001) orientation was identified on which mechanical loading was applied.

### Nanoindentation Experiments

The (0001) grain was subject to repeated nanoindention (NI), performed in a continuous stiffness mode using a 21 μm hemispherical tip; the targeted strain rate was set to 0.1 s^−1^. The NI experiment consisted of 50 loading cycles, on the same location, between a maximum load of 500 mN and minimum of 5 mN, followed by a series of nested cycles consisting of 20–100% of the maximum load shown in supplementary information Fig. 1a. Note the spontaneously and fully reversible stress vs. a/R loops, where a is the contact radius and R is the indenter radius.

### Transmission electron microscopy

TEM lamellae of the indented area were prepared using a FEI DB235 dual-beam focused ion beam (FIB) via a typical *in situ* lift-out procedure. The samples were thinned to electron transparency using a 5kV ion-beam to prevent ion beam damage. Cross-sectional TEM analysis was performed on a JEOL 2100 LaB_6_ equipped with a high-resolution objective-lens pole piece. The GPA analysis was performed using *g* = (0001) and (11
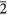
0).

## Additional Information

**How to cite this article**: Gruber, J. *et al*. Evidence for Bulk Ripplocations in Layered Solids. *Sci. Rep.* **6**, 33451; doi: 10.1038/srep33451 (2016).

## Supplementary Material

Supplementary Information

Supplementary Movie 1

Supplementary Movie 2

Supplementary Movie 3

## Figures and Tables

**Figure 2:**
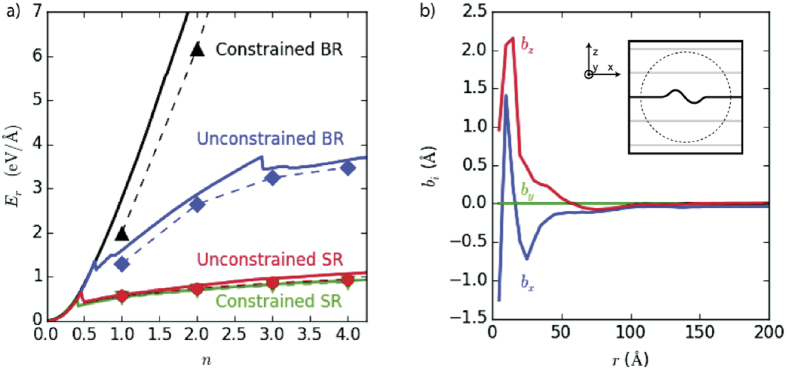
(**a**) Functional dependence of energy per unit length, E_r_, on n for four configurations explored in graphite at 10 K. Dashed and solid lines trace the metastable pathways taken during deformation; the symbols represent minimized periodic structures. Fits of E = n^α^ for surface ripplocations are shown by red dashed lines. Solid black line represents linear in-plane elastic response. (**b**) Burgers vector, **b**_i_ determined from Landau-Lifshitz integral as a function of distance, r, from the core of the BR in graphite; roughly 40 Å from the core, **b**_i_ ≈ 0.

**Figure 3:**
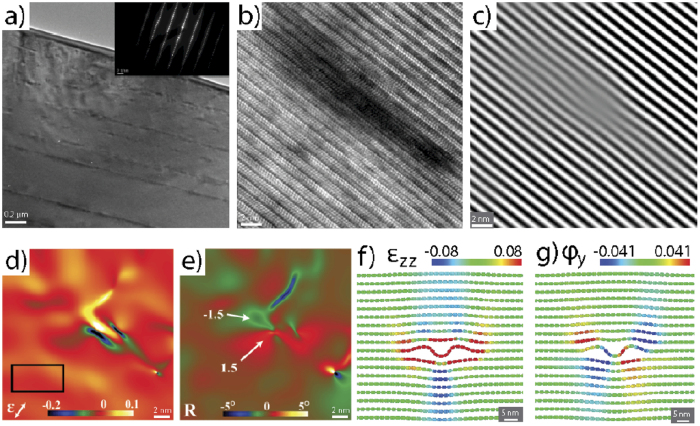
TEM and strain analysis of Ti_3_SiC_2_: (**a**) typical TEM image of an indented Ti_3_SiC_2_ grain along [0001], showing a high defect density-inset shows diffraction pattern, (**b**) high resolution TEM image showing an individual ripplocation; (**c**) IFFT of the same region shown in (**b**) where distortion of the basal planes is clearly visible. Geometric phase analysis is leveraged within this area to produce, (**d**) c-strain, ɛ_zz_, and, (**e**) basal plane lattice micro-rotation maps. The black box in (**d**) denotes the reference lattice for GPA analysis. These are similar to calculated fields of, (**f**) ɛ_zz_ and, (**g**) micro-rotation in graphite determined from our atomistic calculations. In all cases the z-direction was normal to the basal planes.

**Figure 4:**
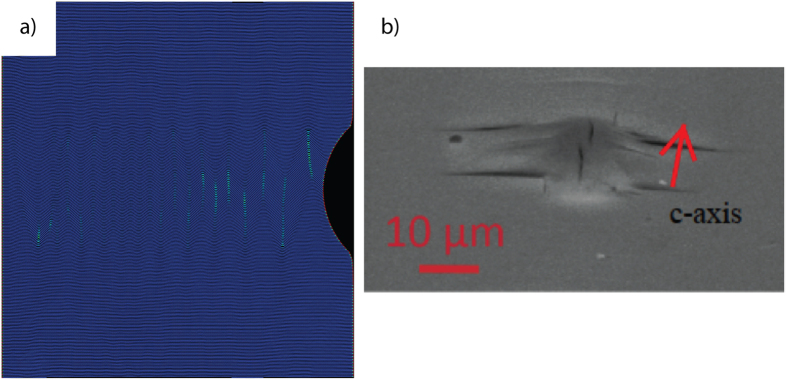
(**a**) Freeze frame from Supplementary Information Movie 3 in which graphitic basal planes are loaded edge-on with a cylindrical indenter from the right. Note spontaneous formation of kink boundaries of opposite signs. At low strains, the process is fully and spontaneously reversible. (**b**) Typical delamination cracks observed when Ti_3_SiC_2_ basal planes are loaded edge-on with a spherical indenter. Such delamination cracks could not nucleate without c-axis strain.
