# Supplementary material for: Evidence for Bulk Ripplocations in Layered Solids
Source: Sci Rep. 2016 Sep 19;6:33451. doi: 10.1038/srep33451 (PMC5027581; doi:10.1038/srep33451)
Supplement: Supplementary Information [file srep33451-s1.pdf]

Evidence for Bulk Ripplocations in Layered Solids
Supplementary Information

Jacob Gruber[1], Andrew C. Lang[1], Justin Griggs[1], Mitra L. Taheri[1], Garritt J. Tucker[1] & Michel W. Barsoum[1]
Department of Materials Science and Engineering,
Drexel University, Philadelphia, PA 19104

Movie Captions:

Movie 1 – Ripplocation Formation
Nucleation of, a) Constrained SR, b) unconstrained SR; c) constrained BR, d) unconstrained BR and, e) their energies as a function of n. Note drops in energy upon nucleation of ripplocations. These energies to do not represent the true ripplocation ground state, but a local energy minimum found by conjugate gradient minimization. Red atoms are free to move while grey atoms are either fixed in space or moved programmatically.

Movie 2 - Ripplocation-Ripplocation
Interaction of two bulk ripplocations, a) of the same polarity and, b) of opposite polarity in the same layer, c) on adjacent layers and, d) on adjacent layers but separated by one layer. In a and b the end result in identical, proving that ripplocations - unlike dislocations - have no polarity. The energy of orientation c is the lowest.

Movie 3 – Simulated Edge-on Nanoindentation
Simulation of the edge-on nanoindentation of a block of graphite with a cylindrical nanoindenter. Periodic boundary conditions are enforced along the y and z axes. Kink bands spontaneously nucleate to accommodate the imposed compressive strain, and fully relax upon the removal of the load.

Supplementary Figures and Captions:

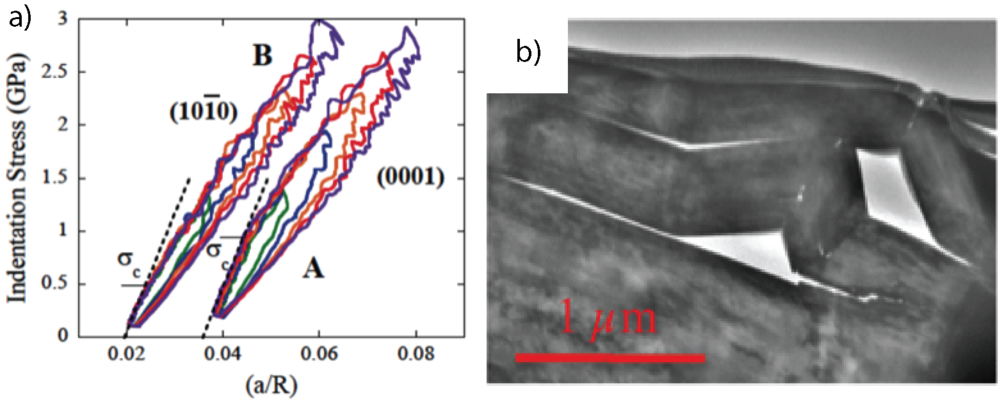

Supplementary Figure 1: KNE Behavior a) Typical nested nanoindentation stress vs. a/R - where *a* is the contact radius and *R* is the indenter diameter - curves obtained when (0001) and (10$\bar{1}$0) planes of Ti_3SiC_2 are indented with a 21 μm spherical nanoindenter. b) Cross-sectional TEM micrograph of Ti_3SiC_2 loaded along [0001], showing nested kink boundaries.

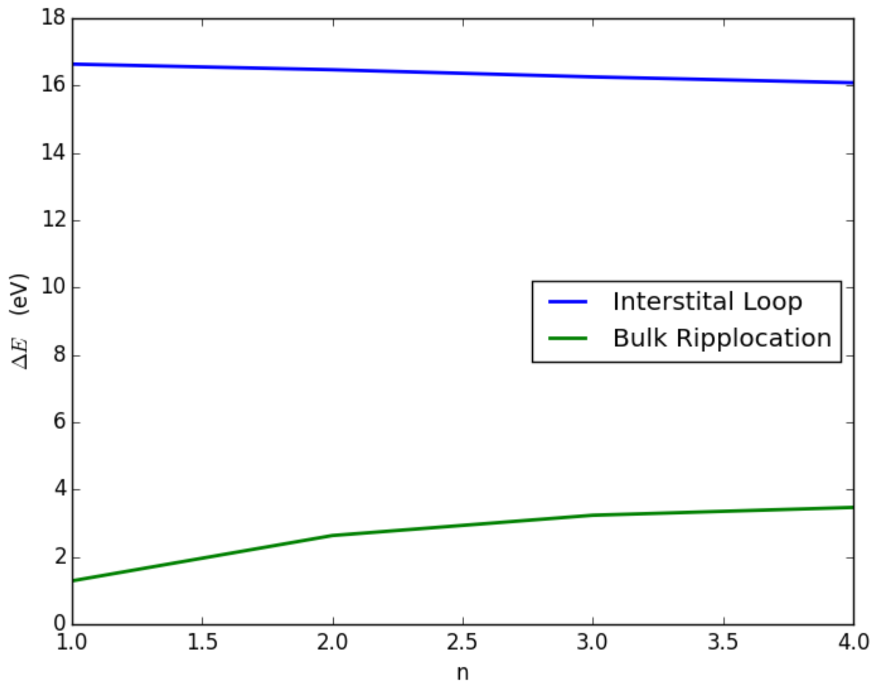

Supplementary Figure 2: Comparison of formation energies of interstitial loops and bulk ripplocations. The cost of the dangling bonds in the interstitial loop outweigh the cost of bent bonds in a bulk ripplocation at small *n*.

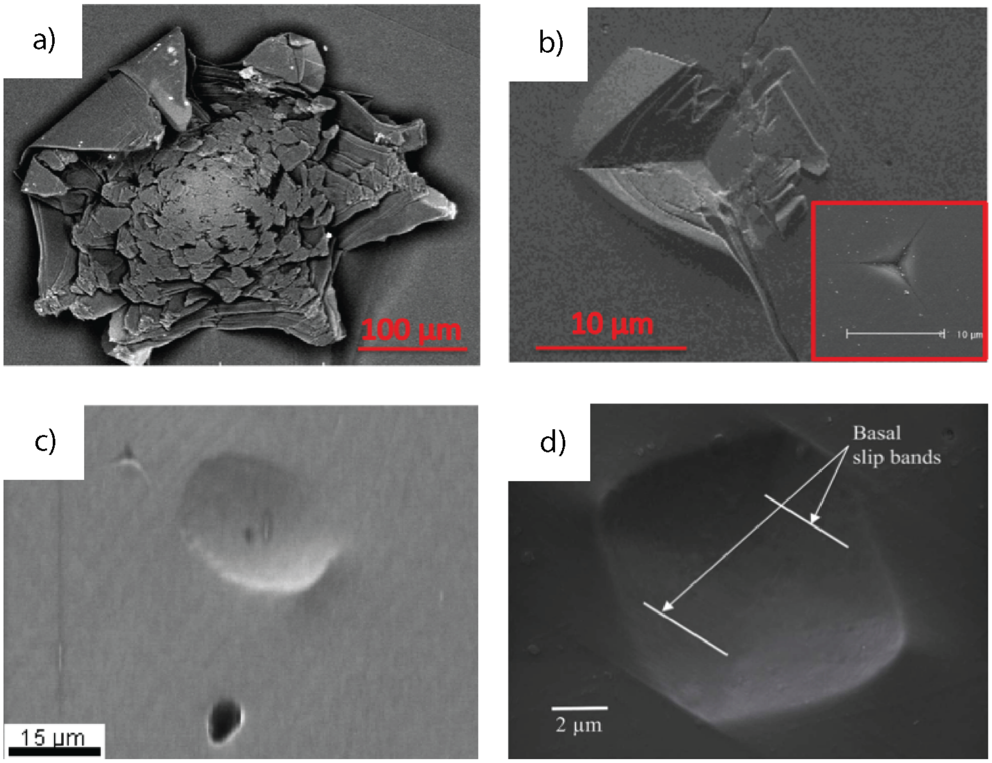

Supplemental Figure 3: SEM micrographs of indentation marks in a) Graphite b) Ti_3SiC_2 with inset SiC c) Zn and d) ZnO. Indentation in graphite, Zn and ZnO was performed using a spherical tip, while indentation of Ti_3SiC_2 and SiC was performed using a Berkovich Tip. Pile-up features are observed in graphite and Ti_3SiC_2, but not in SiC, Zn and ZnO.

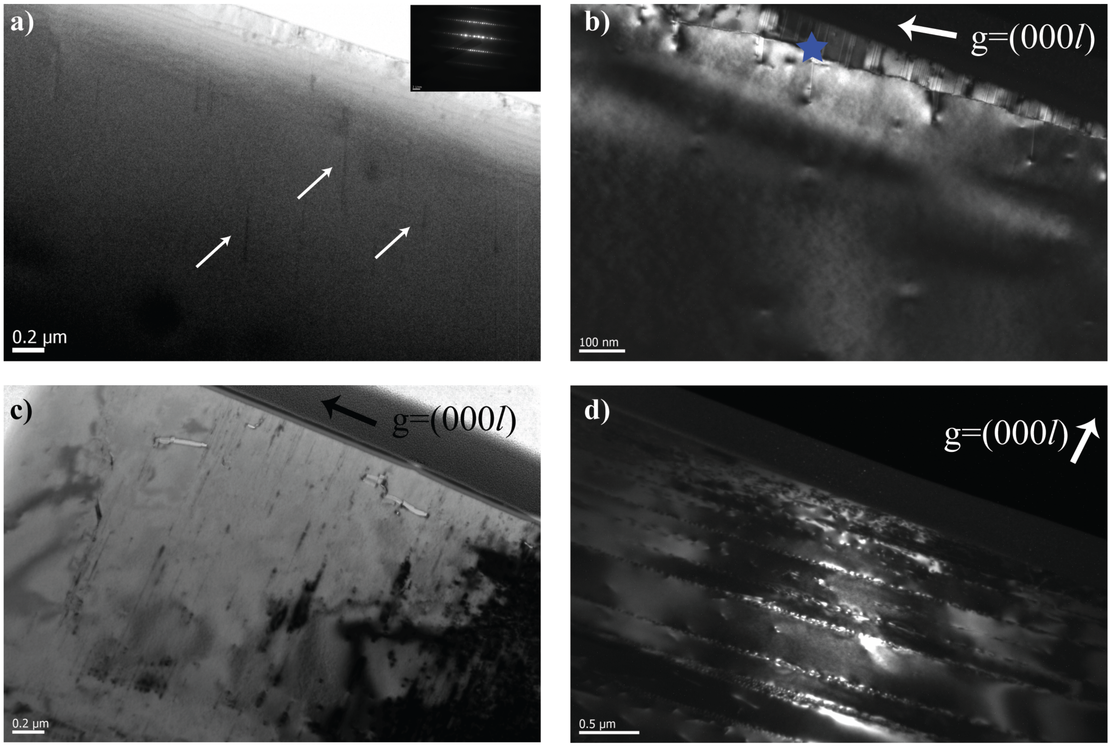

Supplementary Figure 4: TEM images of Ti_3SiC_2. a) Bright-field TEM image away from indented region. b) weak-beam dark-field TEM image of the same area at slightly higher magnification. Note the lack of defects. The area denoted by a blue star in b) is surface damage from mechanical preparation prior to TEM analysis and should be disregarded. c) TEM image of Ti_3SiC_2 grain indented with a spherical indenter parallel to the basal planes near the indented region showing mottled contrast; d) weak-beam dark field TEM image of Ti_3SiC_2 indented normal to the basal planes. Bright mottled contrast is similar to that obtained by Noe and Veblen. These mottled areas were chosen for HRTEM analysis. G-vectors are noted in individual images.
